# First Trimester Typhoid Fever with Vertical Transmission of *Salmonella* Typhi, an Intracellular Organism

**DOI:** 10.1155/2013/973297

**Published:** 2013-12-29

**Authors:** Marguerite B. Vigliani, Anna I. Bakardjiev

**Affiliations:** ^1^Department of Obstetrics and Gynecology, Warren Alpert Medical School of Brown University, East Providence, RI 02914-5300, USA; ^2^Department of Pediatrics, Microbial Pathogenesis and Host Defense Program, University of California, San Francisco, CA 94143-0654, USA

## Abstract

We report a case in which placental abruption occurred at 16 weeks following first trimester diagnosis and treatment for typhoid fever. Unexpectedly *Salmonella enterica* serovar Typhi (*S.* Typhi) was found in fetal tissues at autopsy. Using information from the murine model of typhoid fever in pregnancy, we draw parallels between *S.* Typhi and *L. monocytogenes* to develop a plausible hypothesis to explain how this organism was able to cross the placenta in the first trimester to cause abruption, inflammation, and expulsion of the fetus and placenta. We hope that this model for understanding placental infections by the hematogenous route helps to raise awareness that organisms not typically associated with TORCH infection can nevertheless cause placental infection and pregnancy loss.

## 1. Introduction

A recent case of typhoid fever in the first trimester of pregnancy stimulated our curiosity about organisms that can cross the placenta in pregnancy. Despite early diagnosis and prompt treatment with appropriate antibiotic therapy, fetal loss occurred at 16 weeks with *Salmonella enterica *serovar Typhi (*S*. Typhi), found in the fetus at autopsy. No one caring for the patient had considered that *S*. Typhi could be one of the “other” pathogens on the TORCH (*Toxoplasma gondii*, other, Rubellavirus, Cytomegalovirus, Herpes Simplex virus) list of pathogens. Other microbes not on the TORCH list can also infect the human placenta and affect the fetus (Table 1) [1]. Interestingly many of these same microbes cause abortion in cattle, sheep, goats, and camelids [2]. These microbes disseminate via the hematogenous route and have at least partially intracellular life cycles [3]. It is important to consider these pathogens as a cause for septic abortion or preterm labor in addition to the pathogens that cause chorioamnionitis via the ascending route (e.g., *Escherichia coli* and *Streptococcus agalactiae*) [4].

## 2. Case

A 23-year-old married HIV-negative Cambodian female developed fevers, chills, nausea, vomiting, headache, and vague abdominal pain while travelling home from Cambodia where she had been visiting relatives. She was admitted to an outside hospital for presumed pyelonephritis because of hematuria. After having received two doses of intravenous antibiotics, it was found that she was pregnant and bleeding, so she was transferred to our institution with a tentative diagnosis of septic abortion.

On admission, she was febrile to 103°F and had vaginal bleeding. A pelvic ultrasound showed a viable intrauterine pregnancy of 11 weeks and 6 days. Her blood pressure was 98/60 and her pulse was 90, but both nurse and lab tech had difficulty drawing blood. A finger stick CBC showed Hb 9.8 g/dL (nl 11.7–16.0) and wbc 7.8 (nl 4.0–11.0). A manual differential on 100 cells showed 17 bands and 20 lymphocytes. The platelets were estimated to be low. Her bilirubin was normal, but albumin was 2.0 gm/dL (nl 3.8–5.0), and transaminases were elevated, SGOT 57 U/L (nl 12–30); SGPT 37 U/L (nl 5–32).

Shortly after admission, she became increasingly weak and obtunded with blood pressures of 70/40. She was transferred to the ICU where she was treated empirically for sepsis with Zosyn and Azithromycin. Her condition stabilized. Within 24 hours, both aerobic and anaerobic blood cultures grew *S. *Typhi, and her antibiotics were switched to Ceftriaxone. She continued to spike fevers to 103°F for seven days while on antibiotics, but ultimately she defervesced and was discharged where she completed a 14-day course of IV Ceftriaxone in accordance with CDC recommendations.

She developed recurrent vaginal bleeding and lower abdominal pain after the antibiotics were completed, but there was good fetal growth by ultrasound and the cervical os remained closed. Followup blood cultures and stool cultures were negative for *S*. Typhi and her blood counts were unremarkable, but vaginal bleeding and lower abdominal pain persisted. At 16 weeks she delivered an 86 gm female fetus in the ER. The heart rate was present at delivery, but absent at 15 minutes.

The products of conception were sent for postmortem examination. Cultures of the placenta were not ordered by the emergency physician. The fetus did not have any congenital anomalies or growth retardation and all measurements were consistent with 16 weeks gestational age. Widespread petechial hemorrhages in many organs were suggestive of recent acute intrauterine stress and hypoxia. Although fetal blood cultures were negative, *S. *Typhi was isolated by culture from the fetal lung, consistent with vertical transmission. The placenta was large for gestational age, consistent with transplacental infection. There was mild acute chorioamnionitis and an adherent blood clot associated with placental infarct, with intra- and intervillous hemorrhage involving 70% of the maternal surface.

## 3. Comment

This case is notable because *Salmonellae* are usually not considered TORCH organisms, making it inscrutable that *S*. Typhi found its way into the fetus despite minimal pathological evidence of chorioamnionitis and despite two weeks of treatment with an antibiotic known to cross the placenta. Regrettably, the emergency physician did not order Gram stains or cultures of the placenta, but it begs the question because *S*. Typhi was found in the fetal lung proving not only that the organism could cross the placenta, but also that it had found a way to evade both maternal immune responses and intravenous antibiotics.

*Salmonellae* are facultative intracellular Gram-negative bacteria that cause disease in a wide range of host species [19]. *S*. Typhi affects only humans and is the causative agent of typhoid fever. Typhoid fever is contracted by drinking water tainted by the feces of infected individuals. Every year, an estimated 21.7 million cases occur, resulting in approximately 217,000 deaths [20]. The highest incidence is in Southeast Asia where poor sanitation and unclean water are rampant. In the United States 450 cases are reported annually, and most have travelled internationally within 6 weeks of the onset of the disease.

Before the antibiotic era, typhoid fever in pregnancy was a well-known and dreaded disease, associated with a 60–80% risk of abortion and premature labor and a maternal mortality of 15% [21]. Since the introduction of antibiotics there have been a few case reports and case series describing typhoid fever in pregnancy [5–18, 22]. The conventional wisdom and the CDC recommendations for treatment are based on a case-control study by Sulaiman which shows that typhoid fever does not affect the outcome of the pregnancy [22]. However, our review of the literature supports the contention by Carles et al. [18] that infection early in pregnancy carries a worse prognosis for the fetus, based on studies that address gestational age at the time of infection (Table 2). Certainly, our anecdotal experience is consistent with this observation.

Most of the information we have about the pathogenesis typhoid fever in pregnancy is derived from experiments with *S. typhimurium*, a serovar that causes gastroenteritis in humans but produces a disseminated disease similar to typhoid fever in mice [23]. Infections of pregnant mice with *S. typhimurium* result in 100% fetal loss and 60% maternal mortality.* S. typhimurium* proliferates in the infected placenta and causes widespread placental necrosis and inflammation leading to fetal death and maternal disease [24, 25]. Interestingly, the inflammatory response triggered by the bacterium appears to be more important for the clinical outcome than the bacterial burden. To wit, infection of the placenta with a mutant strain of *S. typhimurium* that is unable to cause inflammation does not induce fetal or maternal mortality despite bacterial burdens similar to wild-type infection [25].

The murine model of *S. typhimurium* thus explains the relationship between bacteria in the placenta, inflammation, placental necrosis, and fetal loss, but it does not explain how the organism breaches the placental barrier. To answer that question, we invite the reader to consider the analogy to *Listeria monocytogenes, *a well-studied enteric organism, which bears similarities to *Salmonella.*

*Listeria monocytogenes* has provided a prototype for understanding placental infection by intracellular organisms via the hematogenous route [3]. *L. monocytogenes* is a ubiquitous facultative intracellular Gram-positive bacterium that causes food-borne disease in humans and other mammals [26]. Infection in pregnancy can result in spontaneous second trimester abortion, preterm labor, and neonatal sepsis or meningitis with mortality rates as high as 50% [27]. The pregnant guinea pig model of listeriosis has shown that the placenta is generally resistant to infection [28]. A mere fraction of the maternal load manages to colonize the placenta, but once infected, even by a single founder bacterium, a clonal infection can start. The placenta becomes a nidus of infection, causing continuous seeding of bacteria to the fetus and to maternal organs. Antibiotics that kill extracellular but not intracellular *L. monocytogenes* demonstrate that the majority of bacteria in the placenta, maternal organs, and blood are inside of host cells.

The decidua is the initial site of placental colonization in experimental models for *L. monocytogenes*, *T. gondii, Chlamydia psittaci*, *Coxiella burnetii, Fusobacterium nucleatum*, and *Brucella abortus *[3]. Since there is no physical barrier between invasive fetal trophoblasts and maternal decidual cells it is not surprising that *L. monocytogenes* can spread from maternal macrophages to invasive fetal trophoblasts. In contrast, syncytiotrophoblasts are very resistant to infection by viral [29, 30], bacterial [31], and protozoan pathogens [32] and are underlain by a continuous basement membrane which acts as an additional physical barrier against pathogen invasion.

*Salmonellae* are a well-known cause of abortion in livestock, resulting in significant economic damages. In humans nontyphoidal *Salmonellae *have been associated with sepsis and early second trimester pregnancy loss, similar to *S*. Typhi in our patient [33–35]. Since *Salmonellae* are intracellular organisms, it is reasonable to speculate that decidual infection might have occurred early in the illness, prior to diagnosis or treatment, during an episode of bacteremia. Our hypothesis is that an abruption occurred at 16 weeks as the result of a delayed but robust host inflammatory response to the continuing presence of the pathogen in the placenta. We know that the organism crossed over into the fetal compartment, and we surmise that the most likely mechanism might have been via infection of fetal invasive trophoblasts in the maternal decidua. There was only minimal chorioamnionitis in the placenta suggestive of hematogenous infection leading primarily to placentitis. Consistent with this hypothesis is that our patient had placental abruption involving 70% of the maternal surface, a finding that parallels the placental necrosis seen in the murine model of pregnancy-associated typhoid fever [24, 25].

By this case report we hope to challenge the prevailing TORCH paradigm. We propose that researchers and clinicians alike consider the hypothesis that any organism with even a partially intracellular lifecycle may potentially infect the placenta via the hematogenous route. There is ample evidence in the literature that various intracellular organisms can travel inside of the immune cells, and that maternal immune cells can be recruited to the fetal implantation site, where extravillous trophoblasts with immune modifications are juxtaposed to maternal decidual cells. Given sufficient invasive and evasive strategies, some intracellular organisms, like *S. *Typhi, may be able to take advantage of these opportunities to cause significant damage to the mother or the fetus.

## Figures and Tables

**Table 1:** Organisms that invade the placenta to cause fetal damage and maternal complications are all intracellular for a portion of their lifecycles.

Bacteria	Parasites	Viruses*
*Brucella *spp. (F)	*Leishmania *spp. (O)	Cytomegalovirus (O)
*Coxiella burnetii *(F)	*Plasmodium falciparum *(O)	Lymphocytic choriomeningitis virus (O)
*Listeria monocytogenes *(F)	*Toxoplasma gondii *(O)	Parvovirus B19 (O)
*Mycobacterium tuberculosis* (F)	*Trypanosoma cruzi* (F)	Rubella virus (O)
*Treponema pallidum* (E)		Varicella zoster virus (O)
*Salmonellae *(F)		

O: obligate intracellular. F: facultative intracellular. E: mainly extracellular, but intracellular is documented.

Many other intracellular organisms including *Babesia* spp., Coxsackie B virus, Japanese Encephalovirus, *Leptospira* spp., *Wuchereria bancrofti*, *Candida* spp., *Pasteurella*, *Shigella*, *Campylobacter*, nontyphoidal *Salmonella* spp. and many gingival bacteria including *Fusobacterium nucleatum* merit further study because of human case reports and/or animal studies.

*Epstein-Barr virus, Hepatitis B virus, HIV, and HSV are transmitted perinatally, but rarely cross the placenta.

**Table 2:** Typhoid Fever in Pregnancy (adapted from Carles with permission).

Author, date	No. of patients	<16 weeks	Fetal losses <16 weeks	Infection >16 weeks	Intrauterine fetal deaths >16 weeks	Neonatal sepsis	Neonatal deaths	Perinatal deaths >16 weeks
Riggall et al., 1974 [5]	7	1	1	6	0	0	0	0
Awadalla et al., 1985 [6]	1	0	0	1	1	0	0	1
Amster et al., 1985 [7]	1	1	1	0	0	0	0	0
Sadan et al., 1986 [8]	2	1	1	1	0	0	0	0
Chin et al., 1986 [9]	3	0	0	3	0	3	0	0
Seoud et al., 1988 [10]	14	2	1	12	0	3	0	0
Dildy et al., 1990 [11]	1	1	0	0	0	0	0	0
Figueroa, 1994 [12]	5	2	1	3	0	0	1	1
Gluck et al., 1994 [13]	1	0	0	1	0	0	0	0
Hedriana et al., 1995 [14]	1	1	1	0	0	0	0	0
Koul et al., 1995 [15]	7	0	0	7	0	0	0	0
Leung et al., 1995 [16]	3	0	0	3	0	0	0	0
Zenilman, 1997 [17]	1	0	0	1	0	0	0	0
Carles et al., 2002 [18]	25	3	1	22	6	2	0	6

Total	72	12	7	60	7	8	1	8
			(58%)					(13%)
